# Square peg in a round hole: re-thinking our approach to evaluating health system strengthening in low-income and middle-income countries

**DOI:** 10.1136/bmjgh-2017-000406

**Published:** 2017-08-19

**Authors:** Josephine Borghi, Zaid Chalabi

**Affiliations:** 1 Department of Global Health and Development, London School of Hygiene & Tropical Medicine, London, UK; 2 Department of Social and Environmental Health Research, London School of Hygiene & Tropical Medicine, London, UK

**Keywords:** Health Systems, Health Systems Evaluation

**Summary box**

- Health system strengthening is an essential step towards achieving universal coverage goals in low-income and middle-income countries (LMICs). However, health systems are complex and health system strengthening initiatives are often introduced with limited understanding of how they will work in practice and the risk of potential adverse events
- Aid flows to health systems in LMICs have increased substantially in the last 15 years. Donors want to know whether their investments in health system strengthening represent value for money relative to disease control programmes and how to optimise the design of such programmes.
- Conventional evaluation methods ignore the complex dynamic nature of health systems and are insufficient to serve donor needs.
- System dynamics and agent-based modelling methods can reflect the complexity of health systems and be used to estimate value for money for health systems investments in LMICs and predict health system response to any stimulus prior to its introduction, including the detection of potential adverse events.
- There has been very limited application of system dynamics and agent-based modelling within the evaluation of health system strengthening initiatives in LMICs, and their future use by researchers is highly recommended.

To function effectively, health systems require adequate financing; an effective workforce; reliable information for decision making; good governance; and available medicines and health technologies to deliver quality services to their populations.^1^ In low-income and middle-income countries (LMICs), health systems are often limited in this respect and fail to provide comprehensive population coverage of quality healthcare interventions. Health system strengthening comprises strategies to improve one or more of the functions of the health system in order to improve access, coverage, quality or efficiency,^1^ and is recognised to be an essential step towards achieving universal coverage goals.^2^ Only when health systems function efficiently and effectively can they deliver services to meet population needs. The health system is recognised to be a complex and adaptive web of relationships and interactions between people, institutions and resources, and the need for a complex adaptive systems approach to their evaluation has been recognised by researchers and policy makers.^3 4^ While the application of systems thinking to LMIC health systems is growing, this remains largely descriptive and qualitative in nature.^5^ In this paper, we begin by describing the growing importance of donor attention to health systems and resulting aid flows. We argue that current research methods are inadequate to accurately address donor needs: to demonstrate value for money for health systems relative to disease control programmes in a way that recognises and reflects the complexity of the health system and avoids reductionism; and to test system strengthening strategies in the lab, to understand how they work and potential adverse events so as to optimise design. We argue that complexity science methods, and in particular system dynamics modelling (SDM) and agent-based modelling (ABM), offer a promising means of effectively operationalising systems thinking to assess and maximise value for money for health systems programmes in LMICs.

In recognition of their importance, aid flows to health systems have been growing. Development assistance to health systems increased from US$ 540 million in 1990 to 2.7 billion in 2015.^6^ Global health initiatives opened up funding streams to support health system strengthening in eligible countries in the mid-2000s. In Global Fund Round 8 grants, for example, almost 40% of funds went to health system strengthening^7^, and around 10% of the Global Alliance for Vaccines and Immunisation funds between 2006 and 2013.^8^ These figures do not reflect the substantial sums that have been allocated by donors to interventions aimed at strengthening specific health system pillars (eg, financing, human resources). For example, results-based financing programmes, consisting of financial incentives for health workers, are operating in more than 30 LMICs, supported by US$ 1.6 billion in low-interest loans from the World Bank and US$ 410 million from the Results Innovation Trust Fund.

Increasingly, donor governments want to know whether their investments are paying off, and how much to invest in health systems relative to specific disease control programmes, and the relative impacts on population health or value for money of each.^9–11^ Although important, this question risks pushing researchers away from a systems thinking perspective and towards isolating, quantifying and comparing the population-level effects of strategies aimed at strengthening a particular part of the system (eg, financing vs governance).^10^ Evaluations of health systems investments usually apply conventional methods to demonstrate programme effectiveness by measuring changes in patient and population outcomes (eg, ref 12 13). This is problematic as it treats the health system as static, one-dimensional, one-directional and linear.^14^ By their nature, health systems are, however, dynamic, multidimensional, non-linear and with feedback loops.^15 16^ The elements of a health system do not all respond simultaneously to a programme because there are inherent time delays associated with the progression of the programme effects through the system. There is communication between each of the health system elements or ‘pillars’ and feedback, the relationship is not unidirectional. Nor do elements operate independently of one another, for example, changes in financing arrangements will inevitably affect governance structures and human resources, which can in turn feedback and influence financing. Finally, the response of the health system is in general non-linear because the overall impact of two independent stimuli is not necessarily additive (the sum of the impacts of the two stimuli considered separately), it can be superadditive or subadditive.

Furthermore, the current emphasis and demand for evidence is focused on evaluation of interventions that have been implemented.^3^ Because strengthening the health system is generally perceived as a good, such programmes are often implemented in hospitals and clinics, impacting directly on health workers and patients, with very limited if any insight or understanding of how they will work and the risk of potential adverse events.^17^ Yet for new drugs, pharmacological testing is required to understand the effect of the drug on the human body (pharmacodynamics), and to determine the required dosage, and monitor adverse events/safety, prior to clinical trials. Only once the mechanism of action is understood are efficacy studies carried out. While systems thinking approaches can be used to inform the design of programmes,^3 18^ typically no formal test of concept is carried out for health systems programmes, partly because their benefit is not questioned, and partly because of the absence of appropriate tools. Yet exploring the potential effects of programmes in the lab and testing responses to potential design changes before implementation would reduce the risk of adverse events in the real world, and further help to optimise design.

There are a variety of complexity science methods that can be used to unpack the complexity of healthcare systems in an LMIC setting, including network analysis, scenario planning and two mathematical modelling methods: SDM and ABM.^19^ Although all these methods can capture the complex static non-linear associations between elements of a complex system, only SDM^20^ and ABM^21 22^ can model dynamic system behaviour such as that observed within health systems.

ABM and SDM are well-known methods for modelling complex sociotechnical dynamic systems, including the dynamic behaviour of an accident and emergency department^23 24^; accountable care organisations^25^; accountable care organisations^25^; policies to make care more affordable^26^; vaccine distribution systems^27^; and neonatal health policies^28^among many others (eg,  29). These models can be used to evaluate and quantify ex ante the response of a health system to any stimulus, including potential adverse events,^30^ be it for a new programme, or additional resources, a sudden disease outbreak or a natural disaster. The models would provide a computational experimental framework for optimising the performance of a health system^23 31 32^ prior to in vivo testing of pilot programmes, and for enhancing the resilience of a health system to potential exogenous disturbances. Models can also be used to inform the design of subsequent empirical evaluations. System dynamics represents a top–down approach where the interest is in modelling the complex macrobehaviour of the system. ABM on the other hand is a bottom–up approach which is ideally suited to model *emergent* complex macrobehaviour which can only be deduced from modelling interactions at the microlevel between the different elements of the system (eg, health workers, healthcare managers and patients). Model structure can be informed by detailed descriptive mapping of processes and relationships between variables based on interviews or workshops and using tools such as causal loop diagrams and stock and flow diagrams in the case of system dynamics, or process mapping).^33^ Empirical data are still needed for model calibration where available. Parameter estimates can also be derived through interviews with key stakeholders, from the literature or from data from other settings with appropriate adjustments. It is also important to carry out comprehensive sensitivity analysis (deterministic or probabilistic) to evaluate the sensitivity of the outcomes of interest to the uncertainty in the parameter estimates. Each modelling approach has its advantages and disadvantages, and the ideal approach for modelling a health system would depend on the specifics of the system and the nature of the programme under consideration, although the two approaches may also complement each other in understanding complex system behaviour.^34^

To date, there has been surprisingly limited application of these methods within (ex ante or ex post) evaluation of health systems interventions in low-income and middle-income settings. In relation to other classical mathematical modelling methods (eg, statistical models), the use of ABM and SDM poses greater challenges to model validation.^35 36^ However, there is always a balance to strike between faithfully representing the real world with its complexity by using ABM and SDM and alternatively using simple modelling approaches that oversimplify system behaviour. These methods offer great potential to add new insights into the relative effectiveness of health system strengthening compared with disease control programmes and to optimise design prior to implementation. We highly recommend their future use by researchers in this field.
